# Global Proteome Changes in the Rat Diaphragm Induced by Endurance Exercise Training

**DOI:** 10.1371/journal.pone.0171007

**Published:** 2017-01-30

**Authors:** Kurt J. Sollanek, Jatin G. Burniston, Andreas N. Kavazis, Aaron B. Morton, Michael P. Wiggs, Bumsoo Ahn, Ashley J. Smuder, Scott K. Powers

**Affiliations:** 1 Department of Applied Physiology and Kinesiology, University of Florida, Gainesville, Florida, United States of America; 2 Research Institute for Sport and Exercise Sciences, Liverpool John Moores University, Liverpool, United Kingdom; 3 School of Kinesiology, Auburn University, Auburn, Alabama, United States of America; Instituto de Investigacion Hospital 12 de Octubre, SPAIN

## Abstract

Mechanical ventilation (MV) is a life-saving intervention for many critically ill patients. Unfortunately, prolonged MV results in the rapid development of diaphragmatic atrophy and weakness. Importantly, endurance exercise training results in a diaphragmatic phenotype that is protected against ventilator-induced diaphragmatic atrophy and weakness. The mechanisms responsible for this exercise-induced protection against ventilator-induced diaphragmatic atrophy remain unknown. Therefore, to investigate exercise-induced changes in diaphragm muscle proteins, we compared the diaphragmatic proteome from sedentary and exercise-trained rats. Specifically, using label-free liquid chromatography-mass spectrometry, we performed a proteomics analysis of both soluble proteins and mitochondrial proteins isolated from diaphragm muscle. The total number of diaphragm proteins profiled in the soluble protein fraction and mitochondrial protein fraction were 813 and 732, respectively. Endurance exercise training significantly (P<0.05, FDR <10%) altered the abundance of 70 proteins in the soluble diaphragm proteome and 25 proteins of the mitochondrial proteome. In particular, key cytoprotective proteins that increased in relative abundance following exercise training included mitochondrial fission process 1 (Mtfp1; MTP18), 3-mercaptopyruvate sulfurtransferase (3MPST), microsomal glutathione S-transferase 3 (Mgst3; GST-III), and heat shock protein 70 kDa protein 1A/1B (HSP70). While these proteins are known to be cytoprotective in several cell types, the cyto-protective roles of these proteins have yet to be fully elucidated in diaphragm muscle fibers. Based upon these important findings, future experiments can now determine which of these diaphragmatic proteins are sufficient and/or required to promote exercise-induced protection against inactivity-induced muscle atrophy.

## Introduction

Maintaining skeletal muscle mass is important for protecting health and sustaining the quality of life. Unfortunately, prolonged periods of muscular inactivity (e.g., limb immobilization, bed rest, or mechanical ventilation) leads to a decrease in muscle mass (i.e., atrophy) and muscle weakness [1]. Of the many forms of disuse muscle atrophy, prolonged mechanical ventilation (MV) is one of the most unique [2]. Indeed, although MV is a lifesaving intervention for critically ill patients, an unintended consequence of this vital intervention is the rapid development of diaphragmatic weakness due to both fiber atrophy and contractile dysfunction; this MV-induced diaphragm weakness is commonly termed “ventilator-induced diaphragm dysfunction” (VIDD) [3, 4]. VIDD is important because diaphragmatic weakness is predicted to be an important contributory factor in the inability to remove patients (i.e. wean) from the ventilator [5]. Difficult weaning leads to prolonged hospitalization along with increased patient morbidity and mortality [6]; therefore, preventing problems in weaning patients from the ventilator is important. Unfortunately, no established clinical therapy exists to prevent VIDD and therefore, developing an intervention to protect against VIDD is imperative.

The explanation as to why prolonged inactivity in the diaphragm results in a more rapid onset of fiber atrophy compared with the time course of disuse atrophy in limb skeletal muscles remains an unsolved mystery [2]. However, while the specific signaling pathways that promote VIDD remain unclear, recent research reveals that increased oxidative stress and mitochondrial dysfunction in diaphragm fibers play a critical role in the etiology of VIDD [2]. Therefore, investigating strategies that prevent oxidative stress and protect against mitochondrial damage could be important to avert VIDD [7–9].

Interestingly, endurance exercise training results in phenotypical changes in cardiac muscle mitochondria that protects these organelles against both oxidative damage and pro-apoptotic stimuli [10]. These changes are significant because exercise-induced “preconditioning” of cardiac mitochondria protects cardiac myocytes against ischemia-reperfusion injury [11]. Similar to cardiac muscle, recent evidence reveals that endurance exercise training, performed prior to MV, protects the diaphragm from VIDD [12]. However, it remains unknown if endurance exercise training alters the mitochondrial phenotype in the diaphragm. This important issue is investigated in the present experiments.

In addition to exercise-induced changes in diaphragm mitochondria, it is also possible that changes in cytosolic proteins could contribute to the exercise-induced protection against VIDD. Note, numerous studies have explored changes in limb skeletal muscle proteins following exercise training [13–26]. However, limited investigations have explored the diaphragm muscle proteome [27–30] and no studies have examined the global proteomic changes that occur in diaphragm muscle following endurance exercise training. It is significant to determine these changes because, as previously stated, the diaphragm is a very unique skeletal muscle compared to limb muscle and thus may have a different response to exercise training. Therefore, the goals of these experiments were twofold. First, we determined if endurance exercise training alters the diaphragmatic mitochondrial phenotype to resist damaging pro-apoptotic stimuli. Second, using the tools of proteomics, we investigated the protein abundance differences in both the soluble and mitochondrial enriched fractions of the diaphragm proteome between sedentary and exercise trained rats. These experiments are important because they form the first required step of identifying the exercise-induced changes in diaphragm muscle proteins that could be responsible for exercise-induced protection of the diaphragm. Subsequently, these candidate proteins can be further vetted to determine potential biological targets for therapeutic intervention to protect against VIDD.

## Materials and Methods

### Experimental Design

This study was carried out in strict accordance with the recommendations in the Guide for the Care and Use of Laboratory Animals of the National Institutes of Health. The protocol was approved by University of Florida Institutional Animal Care and Use Committee (protocol #201304036). Adult Sprague-Dawley (female) rats (~5 mo old) were randomly assigned to one of two experimental groups: sedentary controls (CON) or endurance exercise training (EX). Throughout the experimental period, all animals were housed on a 12:12 hour reverse light-dark cycle and provided rat chow and water ad libitum.

### Exercise Training Protocol

Animals assigned to the EX group performed an endurance exercise training protocol, which has previously been shown to confer a protective phenotype to the diaphragm muscle [12]. Moreover, this protocol was chosen based on previous work from our laboratory as well as preliminary experiments, indicating that this exercise intensity/duration leads to diaphragmatic adaptations in both antioxidants and heat shock proteins [12]. The protocol consisted of a progressive five day habituation period of treadmill running (10, 20, 30, 40, 50 minutes∙day^-1^ on days 1–5, respectively). This was followed by 2 days of rest and then 5 days of running for 60 minutes∙day^-1^. The animals then rested for another 2 days, followed by a final 5 days of training at 60 minutes∙day^-1^. In total, the EX animals completed 10 days of treadmill exercise for 60 minutes∙day^-1^ at 30 meters/minute, 0% grade, which is an exercise intensity that represents approximately ~70% peak VO_2_ for the animals [31]. Note that during our previous investigation, animals were subjected to mechanical ventilation ~24 hours after their last exercise bout [12]. Therefore, the animals used in the current experiments were similarly sacrificed ~24 hours after their last exercise session.

### Tissue Harvesting

Animals in the CON (n = 16) and EX (n = 16) groups were anesthetized and euthanized with an intraperitoneal (IP) injection of sodium pentobarbital (60 mg/kg body weight). Upon reaching a surgical plane of anesthesia, the diaphragm muscle was rapidly dissected and processed as follows. Additionally, the heart was excised from the animals to confirm death. Eight animals in each group (i.e., CON, n = 8; EX, n = 8) had the costal diaphragm removed and used for mitochondrial isolation as described below. From the remaining animals in each group (CON, n = 8; EX, n = 8), the entire costal diaphragm was removed, immediately frozen in liquid nitrogen, and stored at minus 80°C for subsequent proteomic analysis.

### Mitochondrial Measures

#### Isolation of mitochondria

Diaphragmatic mitochondria were isolated from the whole costal diaphragm (approximately 500 mg wet weight) using the methods of Makinen and Lee [32] with minor modifications as previously described [33].

#### Mitochondrial respiration

Mitochondrial oxygen consumption was measured using previously described techniques [33]. Maximal ADP-stimulated respiration (state 3) and was obtained using complex I substrates (i.e., 2mM pyruvate and 2mM malate) in the presence of 0.25 mM ADP and state 4 respiration was recorded following the complete phosphorylation of ADP. Thereafter, the respiratory control ratio (RCR) was calculated as the quotient of state 3 and state 4 respiration.

#### Mitochondrial permeability transition pore (mtPTP) assessment

mtPTP opening is facilitated by increased concentrations of calcium and/or oxidative stress, which leads to mitochondrial swelling, outer membrane rupture, and release of proapoptotic factors [34]. Using previously described techniques [10, 34], the assessment of mtPTP opening was accomplished by monitoring the decrease in light scattering associated with mitochondrial swelling at 540 nm. Isolated diaphragmatic mitochondria were treated with 400 μM CaCl_2_ and 75 μM tert-butyl hydroperoxide. Subsequently, the decrease in absorbance was monitored through a spectrophotometer for 30 mins. The major dependent variables measured for this assessment were maximal rate of pore opening (*V*_max_) and time to reach *V*_max_.

### Isolation of Diaphragm Proteins for Proteomics Analysis

#### Trypsin digest of isolated mitochondria

Aliquots (~100 μg protein) of isolated mitochondria were subjected to three freeze-thaw cycles to create mitochondrial suspensions. Samples were washed with ~200 volumes of 0.1% (w/v) Rapigest SF (Waters; Milford, MA, USA) in 50 mM ammonium bicarbonate using spin columns with 5 kDa molecular weight filters. Samples were adjusted to a final volume of 75 μl in 0.1% (w/v) Rapigest SF (Waters; Milford, MA, USA) in 50 mM ammonium bicarbonate and incubated at 80°C for 15 min. DTT was added (final concentration 1 mM) and incubated at 60°C for 15 min followed by incubation whilst protected from light in the presence of 5 mM iodoacetamide at 4°C. Sequencing grade trypsin (Promega; Madison, WI, USA) was added at a protein ratio of 1:50 and digestion allowed to proceed at 37°C overnight. Digestion was terminated by the addition of 2 μL concentrated TFA and peptide solutions were cleared by centrifugation at 15,000 g for 15 min.

#### Trypsin digest of whole diaphragm muscle

Diaphragm muscles were pulverized in liquid nitrogen then homogenized on ice in 7 volumes of 1% Triton X-100, 50 mM Tris pH 7.4 containing Complete™ protease inhibitor (Roche Diagnostics, Lewes, UK). Samples were incubated on ice for 15 min then centrifuged at 1,000 g, 4°C for 5 min. Supernatants were precipitated in acetone and resuspended in Lysis Buffer: 7 M urea, 2 M thiourea, 4% (w/v) CHAPS, 30 mM Tris, containing Complete™ protease inhibitor (Roche Diagnostics, Lewes, UK). After clearing by centrifugation (12,000 g, 4°C for 45 min) protein concentrations were measured using the Bradford assay (Sigma, Poole, Dorset, UK) and each sample adjusted to 5 μg∙μL^−1^. Aliquots containing 100 μg protein were precipitated in 5 volumes of acetone for 1 hour at −20°C. Pellets were resuspended in 0.1% (w/v) Rapigest SF (Waters; Milford, MA, USA) in 50 mM ammonium bicarbonate and incubated at 80°C for 15 min. DTT was added (final concentration 1 mM) and incubated at 60°C for 15 min followed by incubation while being protected from light in the presence of 5 mM iodoacetamide at 4°C. Sequencing grade trypsin (Promega; Madison, WI, USA) was added at a protein ratio of 1:50 and digestion allowed to proceed at 37°C overnight. Digestion was terminated by the addition of 2 μL concentrated TFA and peptide solutions were cleared by centrifugation at 15,000 g for 15 min.

#### Label-free liquid chromatography-mass spectrometry (LC-MS) analysis

Label-free profiling has been successfully used to resolve a large number of skeletal muscle proteins and we used this approach to determine differences in protein abundance in both the soluble and mitochondrial fractions of the diaphragm proteome between sedentary and exercise trained rodents [35, 36]. Data-dependent label-free analysis was performed using an Ultimate 3000 RSLCTM nano system (Thermo Scientific) coupled to a QExactive^TM^ mass spectrometer (Thermo Scientific). The sample (5 μL corresponding to 200 ng of protein) was loaded onto the trapping column (Thermo Scientific, PepMap100, C18, 75 μm X 20 mm), using partial loop injection, for 7 minutes at a flow rate of 4 μL/min with 0.1% (v/v) TFA. The sample was resolved on the analytical column (Easy-Spray C18 75 μm x 500 mm 2 μm column) using a gradient of 97% A (0.1% formic acid) 3% B (99.9% ACN 0.1% formic acid) to 60% A 40% B over 90 min at a flow rate of 300 nL/min. The data-dependent program used for data acquisition consisted of a 70,000 resolution full-scan MS scan (AGC set to 10^6^ ions with a maximum fill time of 250 ms) the 10 most abundant peaks were selected for MS/MS using a 17,000 resolution scan (AGC set to 5 X 10^4^ ions with a maximum fill time of 250 ms) with an ion selection window of 3 m/z and a normalized collision energy of 30. To avoid repeated selection of peptides for MS/MS the program used a 30 s dynamic exclusion window.

#### Protein search database and statistical analysis

MS spectra were aligned using Progenesis QI for Proteomics (Nonlinear Dynamics Ltd, Newcastle upon Tyne, UK). Prominent ion features (approximately 1200 per chromatogram) were used as vectors to match each dataset to a common reference chromatogram. An analysis window of 9 min—99 min and 200 m/z—2000 m/z was selected encompassing charge states of <+8. Peak lists were searched against the Uniprot database (date 2012 restricted to “Rattus” (35,808 sequences). The enzyme specificity was trypsin allowing one missed cleavage, carbamidomethyl of cysteine (fixed) and oxidation of methionine (variable). The instrument type was ESI-FTICR, mass tolerances were 10 ppm for peptides and 0.01 Da for fragment ions. The Mascot output (xml format), restricted to non-homologous protein identifications (false discovery rate <1%) was recombined with MS profile data in Progenesis QI for Proteomics. Peptide features with MOWSE scores <34 (MudPIT scoring) were excluded and proteins with less than 3 unique peptides were deleted.

Log transformed MS data were normalized by inter-sample abundance ratio and used to investigate differences in expression between CON and EX groups by one-way analysis of variance. Values are presented as means ± standard deviation (SD). To control false discovery rate (FDR), P-value distributions were used to calculate q-values and a criterion FDR of <10% was set. This statistical approach considers the biological variation across each protein and is, therefore, more sophisticated than arbitrarily implementing a threshold based on fold-change.

Functional annotation was conducted using the Database for Annotation, Visualization and Integrated Discovery [DAVID; [37]]. Over-representation of gene ontology (GO) classes: cellular component (CC), biological process (BP) and molecular function (MF) was investigated. Association of proteins with pathways of the Kyoto Encyclopedia of Genes and Genomes [KEGG; [38]] was also assessed.

### Western Blot Analysis

Western blots were performed on both soluble proteins and proteins from the mitochondrial enriched fraction to confirm changes in select diaphragm protein abundance identified by LS-MS analysis. Furthermore, prior to beginning these experiments, we identified several low abundance muscle proteins with cytoprotective properties that are likely below the limits of detection using LS-MS analysis: mitochondrial heat shock protein 70, Sirtuin 3, superoxide dismutase 1, superoxide dismutase 2 and glutathione reductase. Therefore, we used western blotting to determine if exercise training altered the abundance of these proteins in the diaphragm, since many of these proteins were previously demonstrated to increase following exercise from our previous investigation [12]. This approach increases our ability to detect exercise-induced changes in small abundance proteins that have cytoprotective potential. Note, diaphragm samples were not pooled, thus diaphragm samples were loaded independently into each well (N = 8 per group).

#### Mitochondrial protein preparation

Mitochondrial fractions were prepared using previously described methods [33] with modifications outlined below. Mitochondrial proteins were separated on precast 4–15% Tris-HCl gels (Bio-Rad Laboratories) via electrophoresis (200 V for 45 min on ice). Proteins were then transferred to PVDF membrane at 100 mA overnight at 4°C.

#### Soluble protein preparation

Preparation of muscle homogenates and western blotting were performed according to standard procedures as described previously [39] with modifications as outlined below. Briefly, diaphragm tissue samples were homogenized 1:10 (mg wt/ μL buffer) in 5 mM Tris/5 mM EDTA (pH 7.5) with a protease inhibitor cocktail used 1:20 (vol/vol; Sigma-Aldrich; St. Louis, MO) and centrifuged at 30,000 *g* for 30 min at 4°C. After collection of the resulting supernatant, protein content was assessed by the method of Bradford (Sigma-Aldrich) and protein concentrations were normalized using Laemmli sample buffer (161–0737, Bio-Rad; Hercules, CA, USA) containing 5% β-mercaptoethanol. Thereupon, proteins from the supernatant fraction of the diaphragm homogenates were separated on precast 4–15% Tris-HCl gels (Bio-Rad Laboratories) via electrophoresis (200 V for 45 min on ice). Proteins were then transferred to PVDF membrane at 100 mA overnight at 4°C.

#### Protein detection

Subsequently, membranes were blocked using Li-COR Blocking Buffer (Li-COR, Lincoln, NE) for 1 hour at room temp. Mitochondrial proteins were probed for with the following primary antibodies: 3-mercaptopyruvate sulfurtransferase (3MPST, HPA001240, Sigma), mitochondrial heat shock protein 70 (mtHSP70, ab6535, Abcam), GrpE protein homolog 1 (Grpel1, sc-242966, Santa Cruz), Sirtuin 3 (SIRT3, ab86671, Cell Signaling), superoxide dismutase 2 (SOD2, sc-30080, Santa Cruz), and glutathione reductase (GR, ab16801, Abcam).Soluble proteins were probed for with the following primary antibodies: heat shock protein 70 (HSP70, ab6535, Abcam) and superoxide dismutase 1 (SOD1) (sc-11407, Santa Cruz). All primary antibodies were diluted in Li-COR blocking buffer at a 1:1000 ratio, with the exception of Grpel1 (1:200 ratio). Incubations were performed overnight at 4°C. Then membranes were washed in TBS-T (3 ˣ 5 min), and incubated in secondary antibody (IR Dye, 1:20,000, LI-COR) for 30 min at room temp, washed again (TBS-T, 3 ˣ 5 min), and rinsed in 1 ˣ TBS. Finally, membranes were scanned using an Odyssey Infrared Imaging System (LI-COR). For mitochondrial proteins, the immunoblot signal of each target protein was normalized to cytochrome c signals (Cyt c, sc-13156, Santa Cruz) and soluble proteins were normalized to ɑ-tubulin signals (12G10, DSHB).

### Statistical Analysis for Mitochondrial Measurements and Western Blots

After establishing normality using the D'Agostino & Pearson omnibus normality test, between groups comparisons were made by a two-tailed t-test, using CON and EX as the two groups. The statistical analysis was performed using GraphPad Prism® (version 6.00 for Windows, GraphPad Software, La Jolla California USA). Significance was established *a priori* at α < 0.05. Values are presented as means ± standard error of the mean (SEM).

## Results

### Changes to Diaphragmatic Mitochondrial Phenotype Following Exercise

Both sub-populations of mitochondria (i.e., subsarcolemmal and intermyofibrillar mitochondria) were collectively isolated from the entire costal region of the diaphragm. The integrity of our isolation protocol was verified by measuring mitochondrial coupling using the respiratory control ratio (RCR; state 3 respiration divided by state 4 respiration; CON: RCR = 6.3±0.2; EX: RCR = 6.4±0.4). Note that these values are within the range obtained from healthy mitochondria [7, 33]; this confirms that the mitochondria used in these experiments were intact and functionally viable. Further, no differences existed between sedentary controls and exercise trained animals in the mitochondrial RCR values.

**Fig 1:**
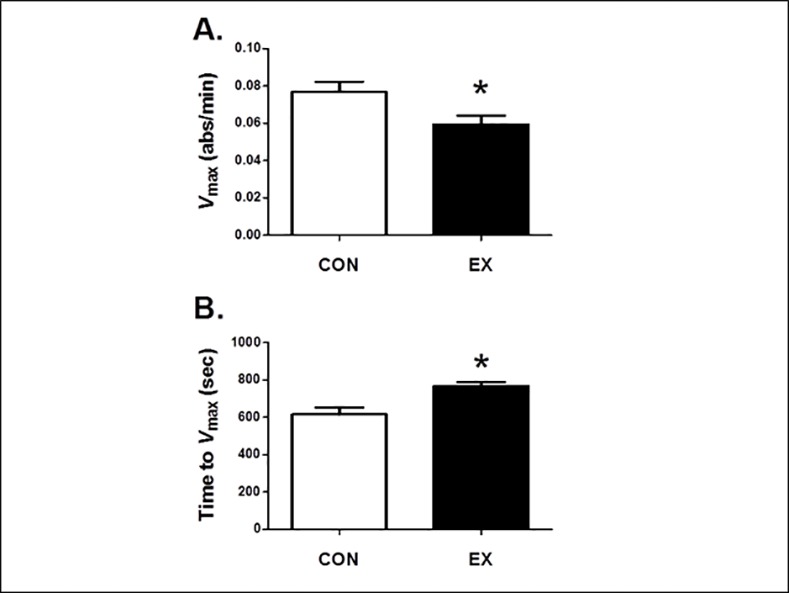
Mitochondrial Transition Pore Kinetics from Isolated Diaphragmatic Mitochondria. A) Maximal rate of pore opening (*V*_*max*_) and B) time to reach *V*_*max*_ after administration of an exogenous high concentration of calcium and *tert*-butyl hydroperoxide (n = 6/group). Values are means ± SEM. *statistically significant difference between groups (p <0.05).

Upon verification of successful mitochondrial isolation, we determined if exercise training altered the mitochondrial phenotype to resist pro-apoptotic stimuli. Specifically, we measured mitochondrial mtPTP opening characteristics by assessing the maximal rate of pore opening (*V*_max_) and the time to reach *V*_max_ during exposure to both calcium and reactive oxygen species (*tert*-butyl hydroperoxide). Our results reveal that endurance exercise training protected mitochondria against pro-apoptotic stimuli as indicated by the finding that exercise training resulted in lower *V*_max_ in mitochondria isolated from EX diaphragm compared with CON (P<0.05) (Fig 1A). Furthermore, isolated diaphragmatic mitochondria from EX animals exhibited a longer time to reach *V*_max_ compared to CON (P< 0.05) (Fig 1B). Combined, these results suggest that mitochondria from exercise-trained animals are more resistant to apoptotic stimuli.

### Identification and Quantification of Relative Protein Abundance in Diaphragm Mitochondria

To investigate the mechanisms responsible for these exercise-induced changes in mitochondrial phenotype, a proteomics analysis was completed on isolated mitochondria. LC-MS/MS analysis of mitochondrial enriched fractions identified 732 (FDR <1%) proteins against the UniProt knowledgebase (UniProtKB). Of these, 302 were annotated as mitochondrial proteins in UniProtKB. When compared against the MitoMiner database of the mitochondrial proteome, 272 of the proteins were present and of those not in the MitoMiner reference set, 55 proteins possessed evidence of mitochondria origin in their GO annotation and therefore, a total 327 known mitochondrial proteins were identified in our analysis. The majority of the proteins that were not associated with the mitochondria were either myofibrillar or proteins associated with membranes of the sarcoplasmic/endoplasmic reticulum, golgi apparatus or lysosome. DAVID analysis of proteins not in mitominer reported enrichment of the following cellular component (CC) classifications: intermediate filament, keratin filament, cytoskeletal part, myofibril, contractile fiber, actin cytoskeleton.

This work is the first label-free profiling of mitochondria enriched from striated muscle and is also the most comprehensive profiling of isolated mitochondria from diaphragm muscle to date. Label-free quantitation was performed using Progenesis QI for proteomics. Differential analysis was performed on 408 proteins that had at least 3 unique peptides. The dynamic range of the label-free analysis spanned 5 orders of magnitude. The most abundant proteins in the mitochondrial enriched fraction included: ATP synthase beta (Relative Abundance [RA] = 5.3E+09), malate dehydrogenase, and ATP synthase alpha. Among the least abundant proteins detected was ornithine carbamoyltransferase (RA = 4.3E+05). We found 25 statistically significant (P <0.05) differences in abundance. Of these, 13 were more abundant in diaphragmatic mitochondria from EX (Table 1) and 12 were more abundant from CON (Table 2). Note, the full data set can be found in the supplementary tables. Calculation of FDR using the Q-value method of Storey and Tibshirani [40] suggests less than 10 percent of these differences may be false discoveries. Functional annotation (DAVID GO) confirmed significant enrichment of the mitochondrial cellular component (CC) but no significant enrichment of BP, MF or KEGG pathways in the list of exercise responsive proteins.

**Table 1:** Proteins Significantly More Abundant in Mitochondrial Enriched Fraction from Diaphragm Muscle from Exercise-Trained (EX) Compared to Sedentary (CON) Animals.

Description	Accession	Gene name	CON (mean ± SD)	EX (mean ± SD)	Difference (%)	Anova (p)
**Energy metabolism**						
Pyruvate dehydrogenase kinase, isoenzyme 4	G3V778	Pdk4	3.3 ± 1.3	4.6 ± 0.9	41	0.028
Pyruvate dehydrogenase E1 component subunit alpha	D4A5G8	Pdha1l1	402.1 ± 32.2	456.7 ± 59.7	14	0.044
Pyruvate dehydrogenase E1 component subunit beta, mitochondrial	P49432	Pdhb	415.6 ± 23	458.2 ± 48.7	10	0.041
ATP synthase-coupling factor 6, mitochondrial	P21571	Atp5j	166.5 ± 20.6	185.9 ± 15.5	12	0.046
**Antioxidants/ apoptosis**						
Aldo-keto reductase family 1, member B10 (Aldose reductase)	Q6AY99	Akr1b10	2.7 ± 0.5	3.4 ± 0.4	25	0.012
GrpE protein homolog 1, mitochondrial	P97576	Grpel1	4.4 ± 0.5	5.4 ± 0.6	22	0.007
Mitochondrial protein 18 kDa	Q5XIG9	Mtfp1	5.6 ± 0.8	6.6 ± 1	18	0.044
3- mercaptopyruvate sulfurtransferase	P97532	MPST	54.8 ± 3.1	63.5 ± 9.3	16	0.026
Mitochondrial carrier homolog 2 (C. elegans)	B0BN52	Mtch2	52.7 ± 3.2	58.9 ± 6.8	12	0.036
**Miscellaneous**						
Prosaposin	Q6P7A4	Psap	1.7 ± 0.2	2 ± 0.3	19	0.020
Ryanodine receptor 1	F1LMY4	Ryr1	18 ± 3.9	21.4 ± 2.1	19	0.038
Aflatoxin B1 aldehyde reductase member 2	Q8CG45	Akr7a2	1 ± 0.1	1.2 ± 0.2	14	0.045
Saccharopine dehydrogenase-like oxidoreductase	Q6AY30	Sccpdh	2.2 ± 0.2	2.5 ± 0.2	10	0.044

Proteins under each sub-heading are ranked (greatest first) by relative differences (percentage difference). Each of the listed proteins exhibited significant (P<0.05) differences in abundance at an FDR of <10%. Description and Database ID relate to the protein name and accession number identified from HDMSE searches of the UniProt Rattus database.

**Table 2:** Proteins Significantly More Abundant in Mitochondrial Enriched Fraction from Diaphragm Muscle from Sedentary (CON) Compared to Exercise-Trained (EX) Animals.

Description	Accession	Gene name	CON (mean ± SD)	EX (mean ± SD)	Difference(%)	Anova(p)
**Cytoskeletal/ myofibrillar**						
Keratin, type II cytoskeletal 1	Q6IMF3	Krt1	15.7 ± 8.0	5.4 ± 1.7	189	0.000
Tropomyosin beta chain	P58775	Tpm2	271.1 ± 122.1	131.5 ± 45.2	106	0.012
Tropomyosin alpha-1 chain 3	P04692	Tpm1	319.2 ± 145.1	158.6 ± 61.8	101	0.016
Keratin, type I cytoskeletal 10	Q6IFW6	Krt10	9 ± 3.4	4.9 ± 1.3	83	0.005
Vinculin	P85972	Vcl	1.3 ± 0.6	0.8 ± 0.3	68	0.037
Actin, alpha skeletal muscle	P68136	Acta1	478 ± 145.6	300.4 ± 91.4	59	0.008
F-actin-capping protein subunit beta	Q5XI32	Capzb	6.1 ± 1.6	3.9 ± 1	53	0.007
Protein Actn2	D3ZCV0	Actn2	2154.9 ± 612.3	1408.9 ± 325.5	53	0.005
F-actin-capping protein subunit alpha-2	Q3T1K5	Capza2	8.1 ± 2.3	5.5 ± 1.4	49	0.010
**Miscellaneous**						
Elongation factor 1-alpha 2	P62632	Eef1a2	4.3 ± 1.4	2.9 ± 0.6	47	0.019
Chaperonin subunit 8 (Theta) (Predicted), isoform CRA_a	D4ACB8	Cct8	1 ± 0.3	0.7 ± 0.1	38	0.018
O-acetyl-ADP-ribose deacetylase	G3V8V6	Macrod1	30.4 ± 3.4	26.2 ± 2.2	16	0.011

Proteins under each sub-heading are ranked (greatest first) by relative differences (percentage difference). Each of the listed proteins exhibited significant (P<0.05) differences in abundance at an FDR of <10%. Description and Database ID relate to the protein name and accession number identified from HDMSE searches of the UniProt Rattus database.

### Identification and Quantification of Relative Protein Abundance from Soluble Diaphragmatic Whole Muscle Homogenate

Note, based upon the methods we used, the soluble proteins analyzed in this investigation contains sarcoplasmic proteins and protein from lysed organelles and do not just represent only cytosolic proteins. LC-MS/MS analysis of soluble proteins isolated from the diaphragm of control and exercise rats identified 1,397 protein groups (FDR <1%). Label-free profiling was performed on 813 protein groups that had 3 or more unique peptides using Progenesis QI for Proteomics. To date, this analysis represents the most comprehensive analysis of diaphragm muscle. The dynamic range of the label-free analysis spanned 6 orders of magnitude. The most abundant protein was creatine kinase M-type (RA = 1.3E+10), least abundant protein detected was 40S ribosomal protein S3 (RA = 2.1E+05).

The normalized abundance of 70 proteins was statistically (P<0.05) different in exercise compared to control samples. Of these, 34 proteins were more abundant in diaphragms from EX (Table 3) and 36 were more abundant from CON (Table 4). Note, the full data set can be found in the supplementary tables. Moreover, 6 of these (non-specific lipid-transfer protein, enoyl-CoA delta isomerase 2, alpha-crystallin B chain, protein flnc, protein acot13 and cytosol aminopeptidase) had q values of <0.1 (i.e. <10% FDR multiple testing correction). Proteins that were more abundant in exercise-trained diaphragm were predominantly mitochondrial proteins involved in mitochondrial protein import, fatty acid catabolism or the electron transport chain.

**Table 3:** Soluble Proteins Significantly More Abundant in Diaphragm Homogenates from Exercise-Trained (EX) Compared to Sedentary (CON) Animals.

Description	Accession	Gene name	CON(mean ± SD)	EX (mean ± SD)	Difference (%)	Anova(p)
**Mitochondrial protein import**						
Mitochondrial import receptor subunit TOM40 homolog	G3V8F5	Tomm40	0.4 ± 0.1	0.5 ± 0.1	25	0.027
Mitochondrial import inner membrane translocase subunit Tim13	P62076	Timm13	3.8 ± 0.5	4.5 ± 0.6	19	0.023
Sorting and assembly machinery component 50 homolog	Q6AXV4	Samm50	3.6 ± 0.4	4.2 ± 0.7	17	0.045
10 kDa heat shock protein, mitochondrial	P26772	Hspe1	37.6 ± 4.3	43 ± 3.6	14	0.015
**Mitochondrial / fatty acid metabolism**						
Non-specific lipid-transfer protein	F1LQ55	Scp2	0.9 ± 0.1	1.4 ± 0.2	53	0.000
Delta(3,5)-Delta(2,4)-dienoyl-CoA isomerase, mitochondrial	Q62651	Ech1	26.5 ± 3.9	36.4 ± 7.8	38	0.006
NADH dehydrogenase (Ubiquinone) 1 beta subcomplex, 7 (Predicted)	D3ZLT1	Ndufb7	7.5 ± 0.5	9.3 ± 1.8	23	0.020
Enoyl-CoA delta isomerase 2, mitochondrial	Q5XIC0	Eci2	7.8 ± 0.6	9.5 ± 0.8	22	0.000
Acyl-CoA thioesterase 2	Q6IMX8	Acot2	21 ± 3	25.4 ± 2.8	21	0.008
Cox7a2l protein	B2RYT5	Cox7a2l	2.9 ± 0.4	3.5 ± 0.6	21	0.028
Protein Acot13	D3ZA93	Acot13	9.8 ± 0.7	11.8 ± 1.1	21	0.000
Protein Hccs	D3ZL85	Hccs	0.6 ± 0.1	0.7 ± 0.1	18	0.041
Platlet glycoprotein 4	F1LQ28	cd36	16.1 ± 1.8	18.9 ± 2.5	17	0.029
Electron transfer flavoprotein subunit beta	Q68FU3	Etfb	97.4 ± 7.7	112.1 ± 12.9	15	0.014
Electron transfer flavoprotein-ubiquinone oxidoreductase, mitochondrial	Q6UPE1	etfdh	51 ± 6.3	58.5 ± 7.1	15	0.033
NADH dehydrogenase (Ubiquinone) 1 beta subcomplex, 11 (Predicted)	D4A7L4	Ndufb11	13.4 ± 1.7	15.5 ± 2.2	15	0.046
Trifunctional enzyme subunit alpha, mitochondrial	Q64428	Hadha	195.1 ± 18.7	223.8 ± 27.1	15	0.027
Long-chain-fatty-acid—CoA ligase 1	P18163	Acsl1	129.8 ± 9.3	147.5 ± 10.9	14	0.003
Dodecenoyl-Coenzyme A delta isomerase (3,2 trans-enoyl-Coenzyme A isomerase)	Q68G41	Eci1	59.9 ± 3.5	68.1 ± 7.3	14	0.010
Protein LOC679794—Cytochrome C	D4A5L9	LOC679794	74.5 ± 7.8	83.6 ± 7.5	12	0.035
Isocitrate dehydrogenase [NAD] subunit alpha, mitochondrial	F1LNF7	Idh3a	75.2 ± 5	83.3 ± 6.4	11	0.015
NADH dehydrogenase (Ubiquinone) flavoprotein 1	Q5XIH3	Ndufv1	102.7 ± 8.6	114.1 ± 9.8	11	0.023
**Secreted Proteins**						
Fibrinopeptide A	P14480	Fgb	2.6 ± 0.4	3.2 ± 0.5	24	0.014
Serine protease inhibitor A3K	P05545	Serpina3k	16.8 ± 2.1	20.6 ± 3.8	23	0.024
Fibrinogen gamma chain	P02680	Fgg	1.9 ± 0.3	2.3 ± 0.3	18	0.027
Galectin-1	P11762	Lgals1	12.6 ± 1.6	13.9 ± 0.7	10	0.048
**Protein folding and antioxidant**						
Heat shock protein 70 kDa protein 1A/1B	Q07439	Hspa1a	19.2 ± 2.8	23.4 ± 4	22	0.038
Protein Mgst3	D4ADS4	Mgst3	4.8 ± 0.8	5.7 ± 0.6	18	0.028
Misc						
PDZ and LIM domain protein 5	Q62920	Pdlim5	1.3 ± 0.3	1.8 ± 0.4	35	0.021
Protein Vwa8	D3ZIN5	Vwa8	2.6 ± 0.5	3.4 ± 0.7	32	0.010
Hydroxysteroid dehydrogenase-like protein 2	Q4V8F9	Hsdl2	18.4 ± 2.5	23 ± 4	25	0.013
Protein Myom1	F1M7T8	Myom1	8.2 ± 1.8	9.8 ± 1	20	0.037
GTP-binding protein SAR1b	Q5HZY2	Sar1b	7.2 ± 0.7	8.5 ± 1.1	19	0.007
Annexin	Q5XI77	Anxa11	10.3 ± 1	11.3 ± 0.8	10	0.036

Proteins under each sub-heading are ranked (greatest first) by relative differences (percentage difference). Each of the listed proteins exhibited significant (P<0.05) differences in abundance at an FDR of <10%. Description and Database ID relate to the protein name and accession number identified from HDMSE searches of the UniProt Rattus database.

**Table 4:** Soluble Proteins Significantly More Abundant in Diaphragm Homogenates from Sedentary (CON) Compared to Exercise-Trained (EX) Animals.

Description	Accession	Gene name	CON(mean ± SD)	EX (mean ± SD)	Difference (%)	Anova (p)
**Heat shock proteins**						
Heat shock protein beta-7	Q9QUK5	hspb7	24.8 ± 5.1	17.7 ± 1.2	40	0.001
Alpha-crystallin B chain	P23928	Cryab	447.6 ± 78	320.8 ± 21.7	39	0.000
Heat shock protein beta-6	P97541	Hspb6	329.8 ± 68.1	254.6 ± 19.7	30	0.005
DnaJ (Hsp40) homolog, subfamily A, member 2	Q5M9H7	Dnaja2	2 ± 0.2	1.6 ± 0.4	26	0.027
Heat shock 27kDa protein 1	G3V913	Hspb1	274.2 ± 52.8	225.9 ± 22.4	21	0.027
Heat shock protein beta-2	O35878	Hspb2	18.2 ± 1	16.7 ± 1	9	0.010
**Signal transduction**						
cAMP-dependent protein kinase type I-alpha regulatory subunit	P09456	Prkar1a	6.5 ± 1	5.4 ± 0.5	21	0.010
Protein kinase, cAMP-dependent, catalytic, alpha	A1L1M0	Prkaca	3.9 ± 0.5	32 ± 0.5	20	0.019
LIM and cysteine-rich domains 1	Q6AYF2	Lmcd1	12.4 ± 2.3	10.6 ± 0.5	17	0.049
Calsequestrin	P19633	Casq1	293.8 ± 15.4	256.4 ± 44.8	15	0.044
Protein Ppp2r1a	Q5XI34	Ppp2r1	19.3 ± 1.7	17.6 ± 1.4	10	0.047
**Protein elongation or degradtion**						
Valine—tRNA ligase	Q04462	Vars	0.9 ± 0.1	0.7 ± 0.2	32	0.031
Elongation factor 1-gamma	Q68FR6	Eef1g	4.5 ± 0.5	3.5 ± 0.7	26	0.016
Protein Nars	F1LPV0	Nars	0.6 ± 0.1	0.5 ± 0.1	23	0.048
Cytosol aminopeptidase	Q68FS4	Lap3	6.2 ± 0.3	5.3 ± 0.4	16	0.000
Ubiquitin carboxyl-terminal hydrolase	D3ZVQ0	Usp5	8.5 ± 0.5	7.9 ± 0.7	9	0.045
**Microfilament structure**						
Gamma-synuclein	Q63544	Sncg	0.6 ± 0.3	0.4 ± 0.1	72	0.006
Protein Xirp1	D4ABA9	LOC100910104	0.8 ± 0.3	0.5 ± 0.1	66	0.019
Protein Flnc	D3ZHA0	Flnc	316.2 ± 50.8	229 ± 20	38	0.000
Gelsolin	Q68FP1	Gsn	7.4 ± 1.3	5.6 ± 0.9	33	0.007
Kelch-like protein 41	Q9ER30	Klhl41	18.2 ± 4.4	13.6 ± 2.9	33	0.022
Filamin alpha	C0JPT7	Flna	1.5 ± 0.3	1.2 ± 0.2	26	0.022
EH domain-containing protein 1	Q641Z6	Ehd1	6.9 ± 0.8	5.9 ± 0.7	16	0.026
Protein Unc45b	D4A8U9	Unc45b	3.7 ± 0.5	3.2 ± 0.4	16	0.035
Tubulin beta-5 chain	P69897	Tubb5	12.5 ± 0.9	10.7 ± 1.2	16	0.006
Cytoplasmic dynein 1 heavy chain 1	P38650	Dync1h1	1.6 ± 0.2	1.4 ± 0.2	14	0.038
**Secreted proteins**						
Haptoglobin	P06866	Hp	1.8 ± 0.6	1.2 ± 0.5	49	0.043
Fetuin-B	Q9Qx79	Fetub	5.7 ± 1.3	4.3 ± 0.9	36	0.014
Inter alpha-trypsin inhibitor, heavy chain 4	Q5EBC0	Itih4	8.9 ± 1.4	6.7 ± 2	33	0.018
Serum albumin	P02770	Alb	3813.9 ± 241.1	3084.1 ± 603.9	24	0.009
Serotransferrin	P12346	Tf	312.4 ± 41.3	261.7 ± 51.8	19	0.039
Alpha-1-macroglobulin	Q63041	A1m	45.8 ± 4.3	39.4 ± 6.3	16	0.027
**Misc.**						
Myosin-11	Q63862	Myh11	0.9 ± 0.5	0.5 ± 0.2	86	0.030
Retinal dehydrogenase 1	P51647	Aldh1a1	7.2 ± 1.6	5.3 ± 1.3	36	0.030
Transmembrane protein 38a (Predicted), isoform CRA_b	G3V9T4	Tmem38a	1.3 ± 0.2	1 ± 0.3	32	0.016
AMP deaminase 1	P10759	Ampd1	27.4 ± 5.2	21.8 ± 5.7	25	0.041

Proteins under each sub-heading are ranked (greatest first) by relative differences (percentage difference). Each of the listed proteins exhibited significant (P<0.05) differences in abundance at an FDR of <10%. Description and Database ID relate to the protein name and accession number identified from HDMSE searches of the UniProt Rattus database.

### Exercise-Induced Changes in Mitochondrial and Cytosolic Proteins Identified by Western Blot

**Fig 2:**
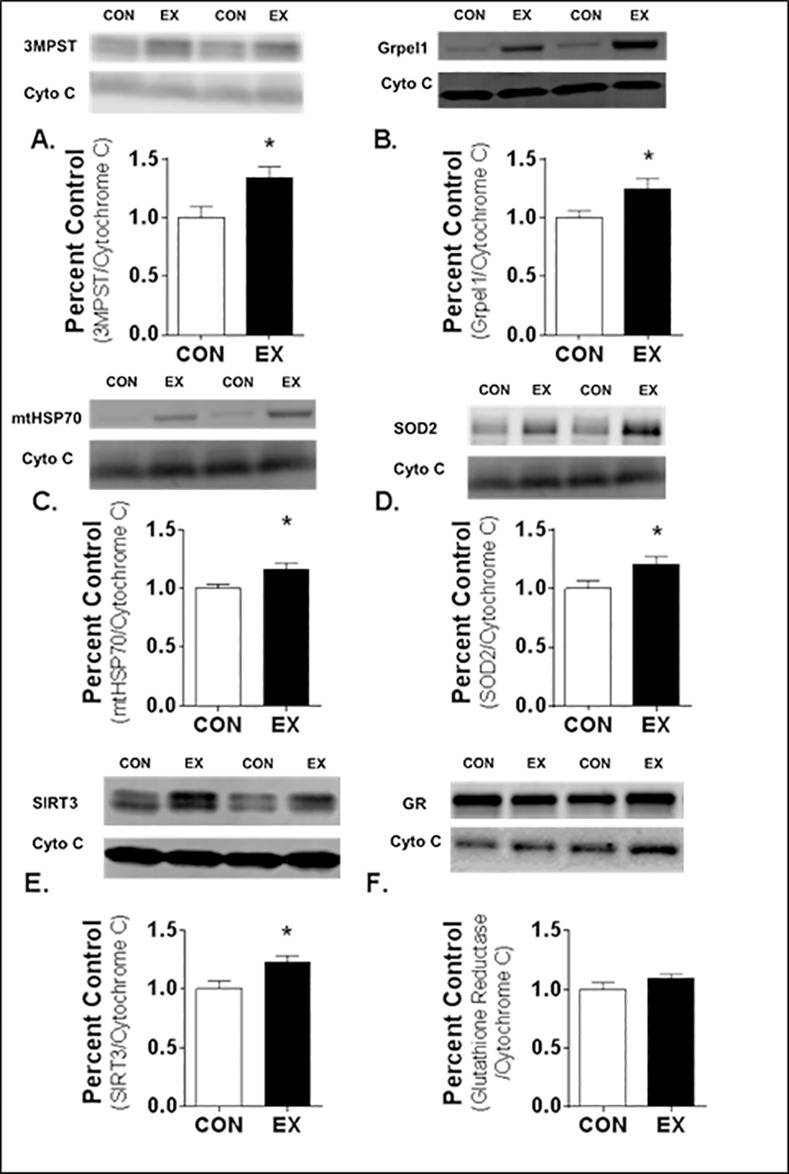
Relative Protein Abundance of Select Proteins in Mitochondria Enriched Fraction from Diaphragm Muscle. A) 3-mercaptopyruvate sulfurtransferase (3MPST), B) GrpE protein homolog 1 (Grpel1), C) mitochondrial heat shock protein 70 (mtHSP70), D) superoxide dismutase 2 (SOD2), E) sirtuin 3 (SIRT3), and F) glutathione reductase (GR). Values are mean percent change compared to control ± SEM and normalized to cytochrome (Cyto) C (N = 8 per group). *statistically significant difference between groups (p <0.05).

**Fig 3:**
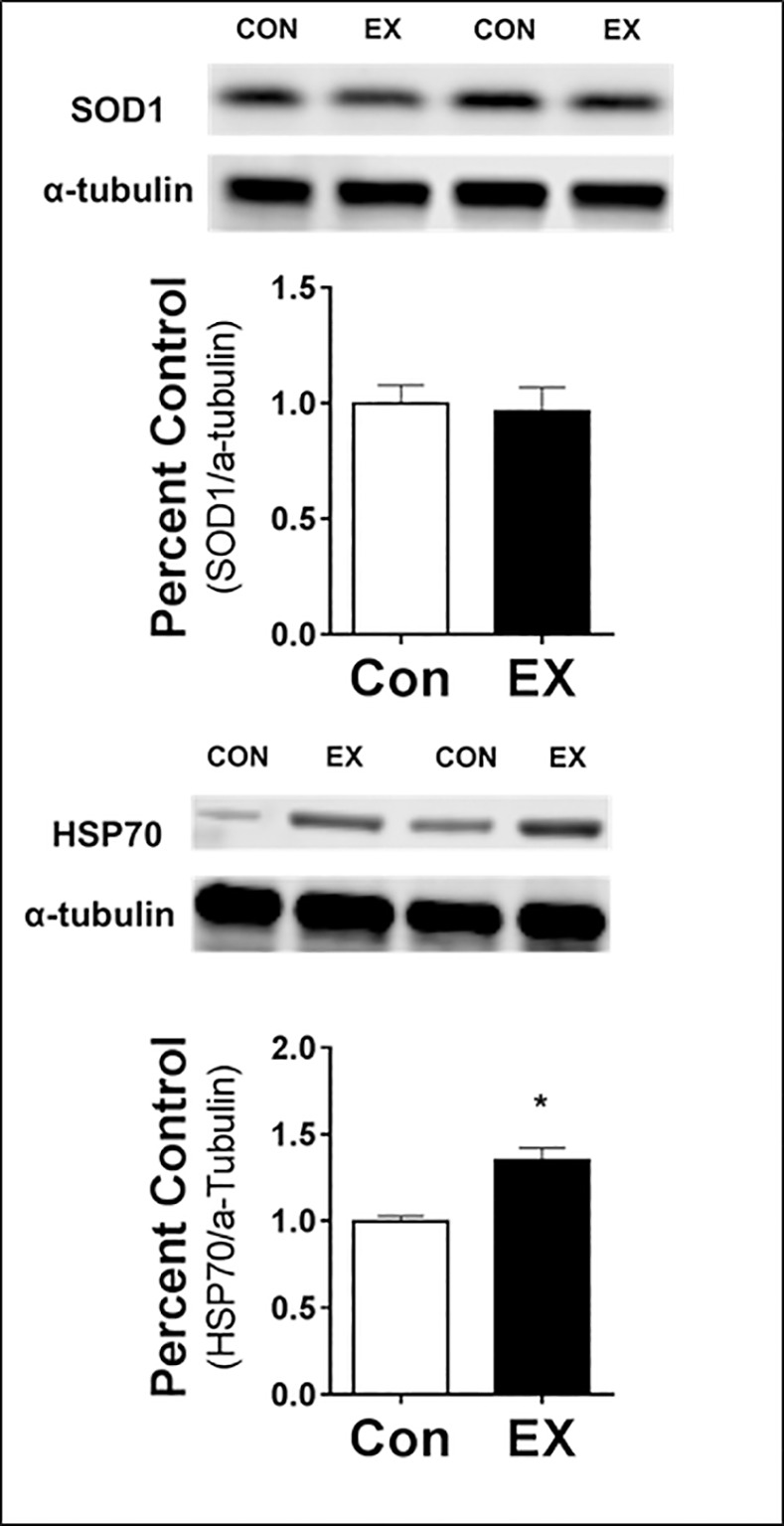
Relative Protein Abundance of Select Proteins in Cytosolic Fraction from Diaphragm Muscle. A) superoxide dismutase 1 (SOD1), and B) heat shock protein 70 (HSP70). Values are mean percent change compared to control ± SEM and normalized to alpha (α)–tubulin (N = 8 per group). *statistically significant difference between groups (p <0.05).

We performed western blot analysis of selected proteins for two purposes: 1) to provide conformational analysis of proteins demonstrated to increase via the proteomics investigation, and; 2) to probe for exercise-induced increases in low abundance proteins that we previously have shown to possess cytoprotective properties and increase with exercise training [12]. Regarding the first purpose, our western blot analysis confirmed the LC-MS/MS data in that exercise training increased diaphragm mitochondrial abundance of 3-mercaptopyruvate sulfurtransferase (3MPST) and GrpE protein homolog 1 (Grpel1) (Fig 2A and 2B) in the mitochondrial enriched fraction. Moreover, western blotting revealed that exercise training significantly increased cytosolic levels of HSP70 (Fig 3B) in the soluble protein fraction. These results confirm the proteomics analysis.

Regarding the second purpose, which was to probe for other cytoprotective proteins found in low abundance in muscle fibers, our western blots revealed that exercise training increased the abundance of mitochondrial heat shock protein 70 (mtHSP70), SOD2, and SIRT3 (Fig 2C, 2D and 2E). In contrast, exercise training did not increase mitochondrial levels of glutathione reductase (Fig 2F). Additionally, the western blot analysis of the soluble fraction from the diaphragm revealed that exercise training did not increase cytosolic levels of SOD1 (Fig 3A).

## Discussion

This study provides the first direct evidence that exercise promotes important adaptations to diaphragmatic mitochondria conferring an apoptotic resistant phenotype. This adaptation was accompanied by robust changes in mitochondrial protein abundance as revealed by a comprehensive proteome profiling of a mitochondrial enriched fraction using label-free proteomics. Moreover, our study reveals numerous other proteins in the diaphragm that are also altered in response to endurance exercise training. To date, this investigation represents the most wide-ranging proteome analysis of diaphragm muscle and is the first investigation to report exercise-induced alterations within both the mitochondrial and detergent-soluble proteome of diaphragm muscle following exercise training. As mentioned previously, these results are important because the diaphragm is the primary muscle of inspiration in all mammals and is a very unique skeletal muscle. It follows that this muscle could have significantly different adaptations to exercise training compared to limb muscles and indeed, our results support this postulate. Most importantly, these investigations have identified several novel proteins as potential candidates for exercise-induced protection. A brief discussion of these key findings follows.

### Exercise Alters Diaphragmatic Mitochondrial Phenotype to Resist Pro-Apoptotic Stimuli

Previous experiments suggest mitochondrial dysfunction plays a key role in the etiology of VIDD [2]. Therefore, the exercise preconditioning effect in the diaphragm may stem from alterations within mitochondria, which affords these organelles protection from pro-apoptotic stimuli occurring during prolonged MV. For example, during prolonged MV, diaphragmatic mitochondria increase their emission of reactive oxygen species during both state 3 and state 4 respiration [33]. This increase in mitochondrial reactive oxygen species production is associated with calcium dysregulation, increases in myonuclear apoptosis, and activation of the proteolyic pathways within the diaphragm [7]. Importantly, our results reveal that diaphragmatic mitochondria undergo exercise-induced biochemical adaptations leading to decreased apoptotic susceptibility. For example, when isolated mitochondria from the diaphragm muscle of EX animals are exposed to an oxidative/calcium challenge, they exhibit a significantly lower absolute *V*_max_ (i.e., rate of pore opening) and a longer time to reach *V*_max_, compared with CON animals (Fig 1A and 1B). Thus, endurance training-induced mitochondrial adaptations afford these organelles protection from the stress of pro-apoptotic stimuli and this adaptation may, in part, contribute to the exercise-induced preconditioning that occurs in the diaphragm.

### Comparison of Proteomics Analysis to Previous Skeletal Muscle Analyses

To explore exercise-induced changes in diaphragmatic proteins, a proteomic analysis of both a mitochondrial enriched fraction and the detergent soluble fraction was undertaken. Several reports have investigated the mitochondrial proteome in limb skeletal muscle [41–46]. Note, however, that the current work is the first investigation to chronicle mitochondrial proteins in response to exercise training within the rat diaphragm. In general, the total number of mitochondrial proteins identified in the diaphragm within the present study are similar to previous reports using limb skeletal muscle [41–46].

Moreover, our LC-MS/MS analysis from the soluble fraction of the diaphragm, identified 1,397 and label-free profiling was performed on 813 proteins. This level of proteome coverage is equivalent to recent work in rat gastrocnemius muscle [35] that profiled 811 proteins including each enzyme of the major energy metabolism pathways in skeletal muscle. More importantly, the current work represents the most comprehensive analysis of diaphragm muscle to date and the first to catalogue the changes that occur in the diaphragm muscle following exercise training.

### Differences in Diaphragmatic Proteins Following Exercise Training

Our global proteomics analysis reveals that endurance exercise alters numerous proteins within the diaphragm. In the present study, we conducted two separate proteomics analyses. The first was completed on a mitochondrial-enriched fraction and the second was performed on the detergent soluble protein fraction of diaphragm muscle to investigate the broader muscle proteome. That is, homogenization of skeletal muscle results in the disruption of mitochondrial membranes and the release of soluble mitochondrial proteins into the supernatant. Therefore, proteomic analysis of the soluble protein fraction of skeletal muscle results in the identification of both cytosolic proteins and proteins from organelles such as mitochondria. The following sections, organized by category of protein function, present a brief summary of the exercise-induced changes in diaphragmatic protein abundance.

#### Mitochondrial proteins identified within soluble fraction

Exercise training increased the abundance of many proteins involved with the bioenergetics pathways; this finding was expected based upon previous results from limb muscle proteome analyses [46]. Specific exercise-induced changes in diaphragm proteins include increases in the constituents of the electron transport chain (e.g., NADH dehydrogenase-ubiquinone-1 beta subcomplex, electron transfer flavoprotein-ubiquinone oxidoreductase, etc) and increases in enzymes for fatty acid metabolism (e.g., long-chain-fatty-acid-CoA ligase 1, Acyl-CoA thioesterase 2, etc.). Because these proteins did not increase in isolated mitochondria, it appears likely that the observed increases in these proteins is linked to the fact that endurance exercise training results in an increased abundance of mitochondria in the exercised muscles [47].

#### Anti-apoptotic proteins

Exercise training increased the abundance of several proteins implicated in the regulation of the stress-induced mitochondrial apoptotic pathway. For example, Aldo-keto reductase family 1, member B10 (Akr1b10) was more abundant in diaphragmatic mitochondria from endurance trained animals. This protein is crucial for cell survival because silencing of Akr1b10 results in caspase 3 mediated apoptosis [48].

Another important mitochondrial protein, mitochondrial fission process 1 (Mtfp1; MTP18), was also increased in mitochondria obtained from the diaphragm of exercise trained animals. Mtfp1 is a downstream target of phosphatidylinostol 3-kinase signaling and is predicted to play a role in mitochondrial health. In particular, knockdown of Mtfp1 results in cytochrome c release from mitochondrial which induces apoptosis [49]; in contrast, over-expression of Mtfp1 results in a punctuate mitochondrial morphology suggesting a functional role of Mtfp1 in maintaining mitochondrial integrity [50]. This finding is intriguing in light of new investigations demonstrating that MV results in altered mitochondrial dynamics [9, 51]. Thus, it is feasible that a portion of the exercise-preconditioning effect may stem from changes in proteins that play key roles in mitochondrial dynamics. Certainly, this hypothesis is worthy of future investigation.

#### Chaperone/Stress proteins

Our results reveal that exercise training results in a greater abundance of GrpE protein homolog 1 (Grpel1) in the mitochondria. This protein is a required component of the PAM complex (presequence translocase-associated motor), a complex required for the translocation of proteins from the inner membrane into the mitochondrial matrix. More specifically, Grpel1 works in conjunction with mitochondrial HSP70 (mtHSP70) to control the binding of mtHSP70 to substrate proteins [52]; this function ultimately supports mtHSP70 activity and function.

A well characterized response of skeletal muscle to exercise training is an increase in chaperone proteins that play key roles in stress adaptation; many of these proteins are family members of the highly conserved family of heat shock proteins (HSPs) [53]. One of the most characterized proteins in this family is the 70 kDa member (HSP72), whose mRNA expression and protein abundance has consistently been shown to increase following endurance exercise training in both limb muscles [54, 55] and the diaphragm [12]. Of note, our proteomics investigation detected a significant exercise-induced increase in cytosolic HSP72 (heat shock protein 70 kDa protein 1A/1B; Hspa1a). This is noteworthy because multiple lines of evidence suggest that increased levels of HSP72 can protect skeletal muscle from disuse atrophy [56–58]. Moreover, two independent recent investigations have demonstrated that increasing HSP72 levels, via heat stress [59] or pharmaceutical intervention [51], prior to MV protected against VIDD. Theoretically, HSP72 can protect against disuse muscle atrophy in several potential ways: 1) HSP72 can maintain mitochondrial integrity by protecting mitochondria against apoptotic stimuli [60–62]; 2) HSP72 can prevent proteolysis by binding to oxidized proteins and assisting in their refolding [56, 63]; and/or 3) HSP72 can inhibit the activity of two transcriptional activators, FoxO3a and NF-κB, which play key roles in the atrophic process [58]. Moreover, in a previous investigation, we observed that exercise training prior to MV results in significant increases in diaphragmatic levels of HSP72 [12]. Future experiments are warranted to determine if exercise-induced increases in HSP72 alone is sufficient to provide protection against VIDD.

#### Antioxidant proteins

Previously, using a targeted analysis, we reported greater antioxidant capacity in the diaphragm of endurance-trained rats [12]. This is significant because administration of exogenous antioxidants prevents VIDD [2]; therefore, it appears likely that increases in antioxidant capacity contribute to the exercise-induced preconditioning effect [12]. Our current findings reveal that exercise promoted an increase in two mitochondrial proteins that possess antioxidant properties. First, exercise increased the mitochondrial abundance of 3-mercaptopyruvate sulfurtransferase (3MPST), which is a novel protein with antioxidant capabilities. In this regard, 3MPST produces hydrogen sulfide which has been shown to be a potent cardioprotective signaling molecule, which is effective in both pre- and post-cardiac conditioning [64, 65]. Specifically, hydrogen sulfide has been shown to act as an antioxidant directly and promote increases in both antioxidant and anti-apoptotic proteins [65, 66].

As previously mentioned, compared to sedentary animals, exercise training elevated the relative abundance of the anti-apoptotic mitochondrial protein, aldo-keto reductase family 1, member B10 (Akr1b10). This protein also has antioxidant capabilities and has been reported to play a role in maintaining redox balance in cells [48, 67]. It follows that exercise-induced increases in this protein could contribute to the cytoprotective effects of exercise training on the diaphragm. However, the role of this and the previously mentioned proteins in contributing to exercise preconditioning is currently unknown and warrants future investigations.

Our western blot analysis also revealed that exercise training resulted in significant increases in mitochondrial levels of both SOD2 and SIRT3 (Fig 2D and 2E). SOD2 is located in the mitochondrial matrix and is responsible for the detoxification of superoxide into oxygen and hydrogen peroxide. Although the increased expression of SOD2 is known to be an important mitochondrial defense mechanism against oxidative stress, post-translational regulation also plays a key role in determining SOD2 activity [68]. In this regard, evidence reveals that SIRT3 plays an important role as a positive allosteric regulator of SOD2 activity. Specifically, SIRT3 decetylates and activates SOD2 to scavenge superoxide [69]. Therefore, our results indicate that exercise training increases mitochondrial antioxidant capacity to eliminate superoxide by increasing the abundance of both SOD2 and SIRT3 in the mitochondria of diaphragm muscle fibers.

Our proteomics analysis of the soluble diaphragmatic proteome revealed an increase in several cytosolic antioxidant proteins following endurance exercise. For example, our results reveal significant increases in a novel antioxidant protein, microsomal glutathione S-transferase 3 (Mgst3; GST-III). In particular, this protein exhibits glutathione-dependent transferase and peroxidase activities and is found in both limb and respiratory muscles [70]. At present, the specific role that exercise-induced increases in Mgst3 plays in protection against VIDD remains unknown and remains an interesting area for future work.

### Limitations of Diaphragmatic Proteome Analysis

Proteomic analysis of skeletal muscle is challenging because muscle contains a large number of proteins and many of these proteins exist in relatively small abundance that challenge detection using current proteomic analysis techniques. Therefore, sub-fractionation of skeletal muscle proteins is often helpful in mining the muscle proteome and the current experiments employed this approach. Furthermore, we also performed western blot analysis to confirm the proteomic analysis (select proteins) to determine if exercise training resulted in an increased amount of specific low abundance muscle proteins with cytoprotective properties.

Note, that our analysis of soluble proteins suggests exercise training decreases diaphragmatic levels of small HSPs including heat shock 27kDa protein 1, heat shock protein beta-6, heat shock protein beta-7, alpha-crystallin B chain, and DNAJ (Hsp40) homolog, subfamily A, member 2. However, several investigations report exercise training causes HSPs to localize to the cytoskeletal/myofibrillar proteins [71], which were removed from our sample. Therefore, because our proteomics analysis specifically focused on detergent soluble (i.e., predominantly cytosolic) proteins, this analysis could fail to detect increases in small HSPs because these proteins were separated-out in the myofibrillar segment of the sample. Therefore, additional experiments examining the myofibrillar proteome following exercise training is a logical next step in the quest to determine which diaphragmatic proteins are altered in response to endurance exercise training.

## Summary and Conclusions

In summary, using state-of-the-art proteomics techniques, this investigation is the first study to catalogue the protein abundance changes that occur in the diaphragm following endurance exercise training and is the most comprehensive profiling of diaphragm muscle to date. Importantly, our results reveal that endurance exercise training leads to numerous changes in diaphragmatic protein expression, many of which have the potential to contribute to the exercise preconditioning effect. For example, our proteomics analysis revealed that endurance exercise training leads to changes in the protein abundance of several diaphragmatic proteins that play roles in bioenergetics (i.e., surrogates for mitochondrial volume), mitochondrial regulation (e.g., import and dynamics), redox regulation (i.e., antioxidant capacity), and stress adaptation (e.g., chaperone proteins). These include increased relative protein abundance of mitochondrial fission process 1 (Mtfp1; MTP18), 3-mercaptopyruvate sulfurtransferase (3MPST), microsomal glutathione S-transferase 3 (Mgst3; GST-III), and heat shock protein 70 kDa protein 1A/1B (HSP70). However, while known to provide protection in other tissues, the cytoprotective roles of many of these proteins have yet to be fully elucidated in diaphragm muscle. Nonetheless, the results of the present investigation have uncovered many new biological targets that have the potential to be pharmacologically manipulated to protect against VIDD. Indeed, the current results provide the platform for future pre-clinical experiments leading to the long-term goal of translation into the clinical setting.

## Supporting Information

S1 Table Proteomic analysis of mitochondrial enriched fraction of diaphragm muscle. Permanent DOI: https://figshare.com/articles/JGB_Progensis_QIP_Diaphragm_analysis_xlsx/4272818/1.
(XLSX)

S2 Table Proteomic analysis of detergent soluble diaphragm muscle proteins. Permanent DOI: https://figshare.com/articles/JGB_Progensis_QIP_Diaphragm_analysis_xlsx/4272818/1.
(XLSX)
